# Supplementary material for: Exchange-torque-induced excitation of perpendicular standing spin waves in nanometer-thick YIG films
Source: Sci Rep. 2018 Apr 10;8:5755. doi: 10.1038/s41598-018-23933-y (PMC5893609; doi:10.1038/s41598-018-23933-y)
Supplement: Supplementary file 1 — Supplementary Information [file 41598_2018_23933_MOESM1_ESM.pdf]

Supplementary Information: Exchange-torque-induced excitation of perpendicular standing spin waves in nanometer-thick YIG films

Huajun Qin[1,*], Sampo J. Hämäläinen[1], and Sebastiaan van Dijken[1,*]

[1]NanoSpin, Department of Applied Physics, Aalto University School of Science, P.O. Box15100, FI-00076 Aalto, Finland
[*]huajun.qin@aalto.fi, sebastiaan.van.dijken@aalto.fi

1. Excitation spectrum of the CPW

We simulated the CPW microwave magnetic field using CST microwave studio software. From the simulations, $\mu_0 h^{rf}$ and the components (in-plane $\mu_0 h_x^{rf}$, $\mu_0 h_y^{rf}$ and out-of-plane $\mu_0 h_z^{rf}$) can be extracted. We define the direction of $\mu_0 h_x^{rf}$ and $\mu_0 h_y^{rf}$ as perpendicular and parallel to the CPW signal line, respectively. The simulations indicate that $\mu_0 h_y^{rf}$ is uniform and $\mu_0 h_z^{rf}$ is much smaller than $\mu_0 h_x^{rf}$. Fourier transformation of $\mu_0 h_x^{rf}$ gives the excitation spectrum of the CPW. The result is shown in Fig. 1. The excitation spectrum is dominated by a narrow peak at wave vector $k \approx 0$. This indicates that the spin wave with almost zero wavevector, so-called ferromagnetic resonance (FMR), can be efficiently excited by such a CPW.

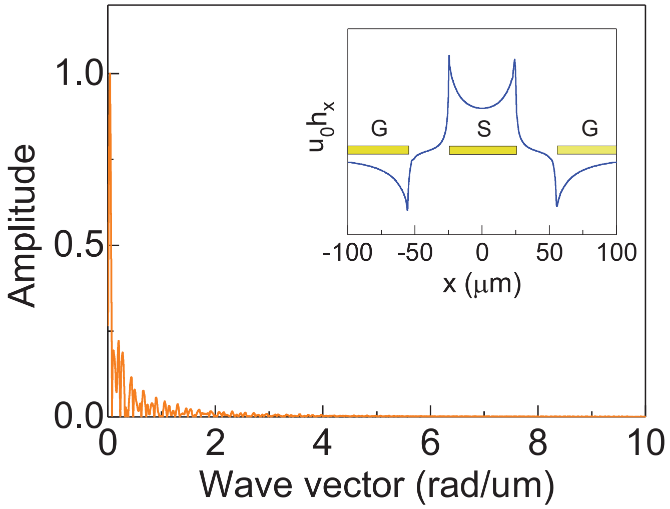

Figure 1. Simulated excitation spectrum of the CPW.

2. Structural characterization of YIG films on GGG(111)

Figure 2 shows high-resolution XRD $\theta - 2\theta$ scans around the (444) reflections of (a) a 295-nm-thick YIG film and (b) a 80-nm-thick YIG film on a GGG(111) substrate. The PLD grown YIG films are epitaxial with an (111) orientation.

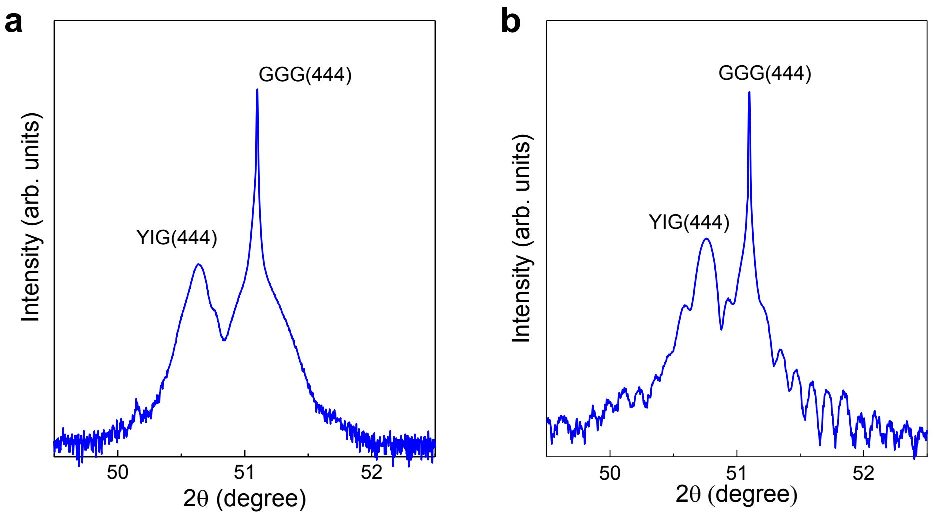

Figure 2. XRD $\theta - 2\theta$ scans of (a) a 295-nm-thick YIG film and (b) a 80-nm-thick YIG film on a GGG(111) substrate.
